# microRNA‐454‐mediated NEDD4‐2/TrkA/cAMP axis in heart failure: Mechanisms and cardioprotective implications

**DOI:** 10.1111/jcmm.16491

**Published:** 2021-05-05

**Authors:** Yaowen Wang, Wei Pan, Xinyu Bai, Xukai Wang, Yan Wang, Yuehui Yin

**Affiliations:** ^1^ Department of Cardiology Chongqing Cardiac Arrhythmias Therapeutic Service Center the Second Affiliated Hospital of Chongqing Medical University Chongqing China; ^2^ Key Laboratory of Basic Pharmacology of Ministry of Education and Joint International Research Laboratory of Ethnomedicine of Ministry of Education Zunyi Medical University Zunyi China; ^3^ Department of Cardiology Institute of Field Surgery Daping Hospital Army Medical University Chongqing China

**Keywords:** apoptosis, cAMP pathway, heart failure, microRNA‐454, NEDD4‐2, oxidative stress, TrkA

## Abstract

The current study aimed to investigate the mechanism by which miR‐454 influences the progression of heart failure (HF) in relation to the neural precursor cell expressed, developmentally downregulated 4‐2 (NEDD4‐2)/tropomyosin receptor kinase A (TrkA)/cyclic adenosine 3',5'‐monophosphate (cAMP) axis. Sprague‐Dawley rats were used to establish a HF animal model *via* ligation of the left anterior descending branch of the coronary artery. The cardiomyocyte H9c2 cells were treated with H_2_O_2_ to stimulate oxidative stress injury in vitro. RT‐qPCR and Western blot assay were subsequently performed to determine the expression patterns of miR‐454, NEDD4‐2, TrkA, apoptosis‐related proteins and cAMP pathway markers. Dual‐luciferase reporter gene assay coupled with co‐immunoprecipitation was performed to elucidate the relationship between miR‐454, NEDD4‐2 and TrkA. Gain‐ or loss‐of‐function experiments as well as rescue experiments were conducted via transient transfection (in vitro) and adenovirus infection (in vivo) to examine their respective functions on H9c2 cell apoptosis and myocardial damage. Our results suggested that miR‐454 was aberrantly downregulated in the context of HF, while evidence was obtained suggesting that it targeted NEDD4‐2 to downregulate NEDD4‐2 in cardiomyocytes. miR‐454 exerted anti‐apoptotic and protective effects on cardiomyocytes through inhibition of NEDD4‐2, while NEDD4‐2 stimulated ubiquitination and degradation of TrkA protein. Furthermore, miR‐454 activated the cAMP pathway via the NEDD4‐2/TrkA axis, which ultimately suppressed cardiomyocyte apoptosis and attenuated myocardial damage. Taken together, the key findings of the current study highlight the cardioprotective role of miR‐454, which is achieved through activation of the cAMP pathway by impairing NEDD4‐2‐induced TrkA ubiquitination.

## 1 INTRODUCTION

As a significant consequence of cardiovascular disease, heart failure (HF) has been reported to affect approximately 26 million people worldwide, many of whom have a dismal prognosis and quality of life.^1^ The elder population is generally more susceptible to HF, which explains the expected increase in incidence in this population owing to the growing aging population as well as increased life expectancy.^2^ HF is characterized by the loss of autonomic balance with the degeneration of vagal activity accompanied by an elevation in sympathetic activity.^3^ HF has been suggested to predominately arise from progressive myocardial disorders coupled with myocardial remodelling.^4^ Cardiomyocyte apoptosis and oxidative stress represent two crucial factors widely documented to participate in the pathogenesis of HF.^5^, ^6^ Thus, further investigation into the molecular mechanisms associated with cardiomyocyte apoptosis and oxidative stress is highly necessary for the development of therapeutic targets for HF.

microRNAs (miRs) represent endogenous non‐coding RNAs (~22 nucleotide) capable of mediating gene expression in a post‐transcriptional manner, some of which have been shown to possess the ability to regulate myocardial remodelling post‐HF by targeting genes.^7^ Of note, miR‐454 has been previously reported to exhibit an aberrant decrease in patients with diastolic dysfunction, an important symptom of cardiovascular disorder.^8^ With miR‐454 as a research focus, we performed prediction of its target genes, based on which neural precursor cell expressed, developmentally downregulated 4‐2 (NEDD4‐2) was predicted to be the target gene of miR‐454. NEDD4‐2 is an ubiquitin E3 ligase capable of disposing target proteins for degradation.^9^ NEDD4‐2 has been previously reported to exert regulatory functions on high‐glucose‐treated rat cardiomyocytes to affect diabetic cardiomyopathy.^10^ Importantly, NEDD4‐2 downregulates voltage‐gated sodium channel Nav1.5 through ubiquitination in the progression of HF.^11^ The activation of NEDD4‐2 is correlated with oxidative stress in hypertension.^12^ Existing literature has highlighted the ubiquitination of tropomyosin receptor kinase A (TrkA) by NEDD4‐2 as a crucial degradation pathway required in the maintenance of neuronal networks.^13^ Depletion of NEDD4‐2 is also reported to activate TrkA signalling.^14^ Likewise, a previous investigation concludes that impaired binding to NEDD4‐2 results in a diminished ubiquitination level of the TrkA neurotrophin receptor in a mouse model, which ultimately influences neuronal survival and their sensitivity to pain.^15^ Intriguingly, TrkA is a receptor of nerve growth factor (NGF), which has been linked to sympathetic neuronal function and neuroanatomy in congestive HF.^16^, ^17^ To our knowledge, TrkA is capable of activating the 3',5'‐cyclic adenosine monophosphate (cAMP) pathway.^18^ cAMP represents a pivotal second messenger that possesses the capacity to modulate heart function by means of contributing to subcellular microdomains in the setting of chronic HF.^19^ A recent study has demonstrated the protective role of this pathway in the cardiac function following HF.^20^ Based on the aforementioned findings and reports, we proposed that miR‐454 may play a regulatory role in HF through mediating the NEDD4‐2/TrkA/cAMP axis. An in vivo HF model in addition to an in vitro oxidative stress model were established in order to identify the effects associated with these mediators on myocardial damage as well as their interaction during the progression of HF.

## 2 MATERIALS AND METHODS

### 2.1 Ethics statement

This study was conducted with the approval of the medical ethics committee of the Daping Hospital, Army Medical University. Signed informed consent documentation was obtained from all participants and their families. The experiments were performed in strict accordance with the ethical guidelines of *Declaration of Helsinki* on human medical research. All experimental procedures involving animals were performed in strict accordance with the Guide for the Care and Use of Laboratory Animals of the National Institutes of Health.

### 2.2 Study subjects

Seventy‐two patients diagnosed with acute myocardial infarction (AMI) complicated with HF at the Department of Cardiology of Daping Hospital, Army Medical University, were enrolled into the current study, including 39 males and 33 females, with a median age of 63 ± 3.9 years from December 2017 to June 2018. As per the Killip classification, there were 25 patients with Killip grade II, 31 patients with grade III and 16 patients adjudged to be grade IV. The Killip classification criteria were as follows: Killip grade II, mild‐to‐moderate HF, third heart sound gallop, persistent sinus tachycardia or other arrhythmias, increased venous pressure and mortality of 10%‐20%; grade III, severe HF, acute pulmonary oedema and mortality of 35%‐40%; and grade IV, cardiogenic shock and mortality of 85%‐95%. The baseline characteristics of all enrolled patients are depicted in Table 1. A total of 72 healthy individuals received coronary angiography at Daping Hospital, Army Medical University, during the same period were randomly selected as the controls, including 37 males and 35 females, with a median age of 58.5 ± 5.4 years. The inclusion criteria of the experimental group as well as the control group were as follows: age <70 years old, no history of chronic diseases such as hypertension, type 2 diabetes, malignant tumour, lung disease, cirrhosis, renal failure, etc A total of 3 mL of peripheral venous blood was collected and placed into ethylenediaminetetracetic acid (EDTA) tubes from all selected individuals who had undergone a period of fasting early in the morning, with the plasma component subsequently separated within 2 hours via centrifugation at 4°C and 3000 g for 10 minutes and stored at −80°C.

**TABLE 1:** Baseline characteristics of 72 patients with AMI complicated with HF

Variable	Category	Overall (n = 72)
Age, mean (SD)	Years	63 ± 3.9
Gender	Female	33
	Male	39
Education	High school	40
	less	32
Marital status	Married	63
	Unmarried	9
Employment status	Employed	42
	Unemployed	30
Blood pressure (mmHg)	Systolic	55 (100‐152)
	Diastolic	41 (60‐87)
Killip class	II	25
	III	31
	IV	16
Underlying heart disease	Coronary artery disease	21
	Cardiomyopathy	18
	Valvular disease	16
	Hypertensive heart disease	10
	Congenital heart disease	4
	Others	3
Body mass index		27 (20‐25)
Heart rate (b.p.m.)		37 (60‐100)
LVEF		
	≤40%	10
	41‐50%	23
	≥50%	39
Duration of disease (h)		23.62 ± 6.49
Co‐medications		
	ACEI or ARB	25
	Beta blockers	21
	CCB	17
	Diuretic agent	9
LVEDV (ml/m^2^)		78.04 ± 13.85
LVESV (ml/m^2^)		25.91 ± 8.63

Abbreviations: AMI, acute myocardial infarction; b.p.m: beat per minute; HF, heart failure; LVEF: left ventricular ejection fraction; SD, standard deviation.

### 2.3 Establishment of HF rat model

Sixty 8‐ to 12‐week‐old male‐specific pathogen‐free grade Sprague‐Dawley rats weighing 200‐300 g were purchased from Chongqing Tengxin Biotechnology Co., Ltd. The Sprague‐Dawley rats were anaesthetized via intraperitoneal injection of 20% (w/v) urethane (0.5 mL/100 g) solution (Cat. No.: 51‐79‐6; Beijing ALB Technology). The rats were placed in a supine position with their limbs and heads fixed on the operating table; the hair on their neck and chest was shaved off, followed by disinfection with iodine and 70% ethanol. The skin of the middle part of the neck was cut open, after which the subcutaneous muscle tissues were surgically removed in a layer‐by‐layer fashion until the trachea was exposed. Endotracheal intubation was performed using intravenous indwelling needles; the inner core was removed and fixed, followed by connection with a small animal ventilator (DW‐2000) with the ventilation volume maintaining at 1.5 mL–2.0 mL/time, with a respiratory rate of 80 breaths/min. The skin, intercostal muscle and pleura were cut open in turn between the fourth and fifth intercostal spaces of the left thoracic cavity in order to open the thoracic cavity. The heart was fully exposed with the pericardium separated. For HF modelling, the left anterior descending (LAD) coronary artery was ligated approximately 2 mm from the lower edge of the left auricle using 8‐0 nylon suture. LAD ligation was deemed to be successful if the anterior wall of the left ventricle near the apex of the heart was grey white or blue purple, accompanied by ventricular wall abnormality and reduced movement contractile force. An identical operation with no ligation was performed in the sham‐operated rats. The rib, muscle and skin were sutured in turn to close the thoracic cavity; iodine and 70% ethanol were subsequently employed for disinfection in turn. The tracheal intubation was removed following recovery of spontaneous respiration; the skin of the neck was sutured and placed back into the cage for 7 days of standard feeding. Twelve rats were sham‐operated, while the remaining 48 rats underwent LAD ligation for modelling purposes. Among the 48 rats after modelling, 12 rats were administered with an injection of 50 μL of sterile phosphate‐buffered saline (PBS) after 30 minutes of LAD ligation, 12 rats received injection with adeno‐associated virus‐NC (AAV‐NC), respectively, in situ after 30 minutes of LAD ligation, and 12 rats received injection with AAV‐miR‐454 (the AAV‐DJ vector was provided by GenePharma) in situ after LAD ligation. The remaining 12 rats were administrated with an injection of AAV‐miR‐454 in situ following LAD ligation, after which they were intraperitoneally injected with cAMP pathway inhibitor H‐89 at a concentration of 10 μmol/L, 30 mg/kg.^20^ The dosage of the AAVs was 1.5 × 10^14^ vector genomes/kg.

### 2.4 Colour Doppler ultrasound

The rats were mildly and continuously anaesthetized with 2.0% isoflurane and fixed in a supine position onto a test table with their limbs tightly attached to electrodes. A Vevo 2100 (Visualsonics) ultrasonic image detector equipped with 13 MHz probes was utilized to scan the rat hearts, with the long‐axis view of the left heart and the short‐axis view of left ventricular papillary muscle captured beside the left sternum. Left ventricular end‐systolic diameter (LVDSD), left ventricular end‐diastolic diameter (LVEDD), ejection fraction (EF) and fractional shortening (FS) were measured and calculated on the short‐axis view of the left ventricular papillary muscle. Heart rate was evaluated by simultaneous electrocardiographic monitoring.

### 2.5 Haematoxylin‐eosin (HE) staining

The rats were euthanized following a 7‐day period of modelling, with their heart tissues collected, paraffin‐embedded and sectioned. HE staining was performed to examine the pathological changes. After the heart tissues were fixed, dehydrated, waxed, sliced (5 μm in thickness) and placed onto slides, the slides were subsequently baked in a 60°C incubator for 30 minutes, dewaxed, immersed in xylene I and II, each for 20 minutes and hydrated. Following immersion in gradient alcohol (100%, 95%, 80% and 70% each for a 5‐minutes period), the slices were stained with haematoxylin for 2‐3 minutes and then immersed in 1% ammonia water for 1‐2 minutes. The slices were immersed in eosin dye for 1 minutes, followed by dehydration in 70%, 80%, 95% and 100% alcohol for 5 minutes, respectively, and then cleared in xylene for 10 minutes. The slices were finally sealed with neutral resin and observed by microscopy.

### 2.6 Masson's Trichrome staining

The paraffin‐embedded heart sections were baked in a 60°C incubator for 30 minutes, cooled naturally at room temperature and dewaxed three times using xylene (5 minutes/time). The sections were then immersed in gradient ethanol (100%, 95%, 80% and 70% for 5 minutes each) in a successive manner, and then soaked in distilled water for 2 minutes to completely hydrate the sections, followed by staining with haematoxylin for 5 minutes. Colour differentiation with 1% hydrochloric acid alcohol was performed for 5 seconds, after which the sections were immersed in 1% ammonia water for 5 seconds. The sections were then dyed with 0.9% (w/v) Ponceau plus 0.1% (w/v) acid fuchsin and 1% (v/v) glacial acetic acid for 5 minutes and then with 2.5% (w/v) phosphotungstic acid plus 2.5% (w/v) phosphomolybdic acid for 7 minutes, and finally with 2.5% (w/v) aniline blue. The sections were subsequently immersed in 1% glacial acetic acid for 2 minutes, in gradient alcohol (80%, 90% and 100% each for 5 minutes) and in xylene for 10 minutes, after which they were observed by microscopy after sealing with neutral resin. To quantify the degree of myocardial fibrosis, stained sections were analysed with Image‐Pro Plus version 6.3 software (Media Cybernetics).

### 2.7 Terminal deoxyribonucleotidyl transferase dUTP nick end labelling (TUNEL) staining

After dewaxing and complete hydration, the sections were incubated with 20 μg/mL of protease K in a 37°C incubator for 25 minutes, and then incubated with PBS‐formulated 3% hydrogen peroxide solution (3% H_2_O_2_ in PBS) at room temperature for 20 minutes to eliminate the endogenous peroxidase in the sections. The samples were stained in accordance with the instructions provided by TUNEL cell apoptosis assay kit (Beyotime). The samples were incubated for 60 minutes under conditions void of light at 37°C with 50 μL of biotin labelling solution. After one PBS or HBSS washes, 0.1‐0.3 mL label reaction termination solution was added in a dropwise fashion onto the samples (with 10‐minutes incubation at room temperature), followed by incubation with 50‐μL Streptavidin‐horseradish peroxidase working solution for 30 minutes at room temperature, and 0.2‐0.5 mL of diaminobenzidine (DAB) developing solution at room temperature for 5‐30 minutes or for an appropriate time based on the developing condition. The sealed sections were observed and photographed under a microscope, followed by tallying using a double‐blind manner. The apoptosis rate was calculated as the percentage of apoptotic nuclei/total number of nuclei.

### 2.8 Reverse transcription‐quantitative polymerase chain reaction (RT‐qPCR)

TRIzol (Invitrogen) methods were employed to extract total RNA from plasma, tissues and cells according to the manufacturer's instructions. Briefly, samples were homogenized with TRIzol, then one‐fifth chloroform was added and vigorously vortexed. After centrifugation at 12 000 × g for 15 minutes at 4°C, the aqueous phase was removed into a new tube and added with an equal volume of isopropano. The centrifugation was repeated, and the pellet was washed with 75% ethanol. Finally, the RNA pellets were air‐dried and resuspended in RNase‐free water. A Nanodrop2000 micro‐ultraviolet spectrophotometer (1011 U, NanoDrop Technologies Inc) was utilized to determine the total RNA concentration and purity. According to the instructions of TaqMan microRNA Assays Reverse Transcription Primer (4427975, Applied Biosystems)/Primescript RT Regent Kit (RR047A, Takara Holdings Inc), RNA was reverse‐transcribed to complementary DNA (cDNA). The primers for miR‐454, NEDD4‐2 and TrkA were designed and synthesized by Takara (Table S1). Using TaqMan multiple real‐time PCR kit (4461882, Thermo Fisher Scientific Inc), real‐time fluorescence qPCR was performed on a qPCR instrument (ABI7500, ABI Company). Glyceraldehyde phosphate dehydrogenase (GAPDH) and U6 were employed as internal controls, with the relative quantitative method ($2^{‐\Delta \Delta C_{t}}$ method) applied for the calculation of the relative expression of the target genes.

### 2.9 Western blot assay

The tissues and cells were lysed, respectively, using radioimmunoprecipitation assay (RIPA) lysis buffer containing phenylmethylsulfonyl fluoride (PMSF) (Beyotime) on ice for 10 minutes. The supernatant was extracted following centrifugation at 14 000 rpm at 4°C for 10 minutes. The protein concentration was determined by bicinchoninic acid (BCA; Pierce) method. Next, the samples were separated using 4% concentrated gel and 10% concentrated gel, and transferred to membranes. After a 1‐hours blockade using 5% skim milk powder, the membranes were incubated overnight at 4°C with primary rabbit antibodies against Bcl‐2‐associated X protein (Bax; ab32503; 1:10 000), cleaved caspase‐3 (ab2302; 1:500), B‐cell lymphoma 2 (Bcl‐2; ab196495; 1:2000), NEDD4‐2 (ab46521; 1:1000) and TrkA (SAB1305370; 1:1000), which were purchased from Sigma, and antibodies against phosphorylated (p)‐AMP‐dependent protein kinase (PKA; PA5‐105515; 1:2000) and p‐cAMP response element‐binding protein (CREB; PA1‐850; 2 µg/mL), which were purchased from Thermo Fisher Scientific Inc The proteins were incubated with rabbit antibody against immunoglobulin G (IgG; 1:1000, Santa Cruz) labelled with horseradish peroxidase at room temperature for 1 hour and colour was developed in the enhanced chemiluminescence reagent (Thermo Fisher Scientific Inc), followed by development and fixation (Bio‐Rad ChemiDoc Imaging system, Bio‐Rad Laboratories). GAPDH (mouse anti‐GAPDH, 1:1000, Santa Cruz Biotechnology) was regarded as an internal reference, while the protein blot images were subsequently analysed using imageJ2x software.

### 2.10 Immunochemistry

The sample was fixed with 10% neutral formalin, embedded in paraffin and sectioned with an ultramicrotome. The sections were deparaffinized with xylene, rehydrated with graded alcohol and incubated with 3% hydrogen peroxide to block endogenous peroxidase activity. The sections were boiled in 10 mmol/L sodium citrate (pH 6.0) for 30 minutes, then blocked in 10% normal goat serum for 15 minutes and incubated with antibodies against TrkA (PA5‐109216, Invitrogen) and NEDD4‐2 (ab46521, Abcam) overnight in a wet room at 4°C. Next day, the sections were washed with PBS and incubated with the secondary antibody for 1 hour at room temperature. The immunoreactivity was detected with DAB kit (Invitrogen).

### 2.11 Cell culture and transfection

The cardiomyocyte H9c2 cell line was obtained from American Type Culture Collection (CRT‐1446, ATCC). The cells were cultured with Dulbecco's modified Eagles Medium (DMEM; Gibco) containing 10% foetal bovine serum (FBS; Gibco), 100 μg/mL streptomycin and 100 U/mL penicillin, and maintained at 37°C under saturated humidity conditions consisting of 95% air and 5% CO_2_. The cells at the logarithmic growth phase were trypsinized and seeded into 6‐well plates (1 × 10^5^ cells per well). Next, 50% cell confluence was identified following 24 hours of conventional culture. A Lipofectamine 2000 (Invitrogen) was used to transiently transfect the cells using mimic‐NC, miR‐454 mimic, inhibitor NC or miR‐454 inhibitor; mimic‐NC + overexpression (oe)‐NC, mimic NC + oe‐NEDD4‐2, miR‐454 mimic + oe‐NC or miR‐454 mimic + oe‐NEDD4‐2; oe‐NC, oe‐NEDD4‐2, small interfering RNA (si)‐NC, si‐NEDD4‐2; mimic NC + si‐NC, miR‐454 mimic + si‐NC, miR‐454 mimic + si‐TrkA (4 μL Lipofectamine 2000 + 2 μg plasmids). The transfected plasmids mimic, inhibitor and AAV were all purchased and synthesized in Sino Biological (Beijing, China). The siRNA sequences employed were as follows: si1‐NEDD4‐2 (5′‐GCCAUCAGUGGCCUAUGUA‐3′), si2‐NEDD4‐2 (5′‐GCAGAAAUACGACUACUUU‐3′) or si3‐NEDD4‐2 (5′‐GGUCCUCAGCUGUUUACAA‐3′); si1‐TrkA (5′‐AUUCAGGUGACUGAGCCGAGGG‐3′), si2‐TrkA (5′‐AAAAACGUCAUCCCCCACUUCC‐3′) or si3‐TrkA (5′‐UUCUUCUCCACUGGGUCUCUUG‐3′). H‐89 (30 μmol/L) was employed to block the cAMP pathway in the cells transfected with miR‐454 mimic, with dimethyl sulfoxide (DMSO) employed as the control. After 6 hours of transfection, the culture medium was renewed; 8 hours later, the cells were collected for subsequent experiments.

### 2.12 Oxidative stress cell model

Upon reaching 80% confluence following plasmid transfection, the H9c2 cells were treated with 200 μm H_2_O_2_ for 12 hours to induce oxidative stress. At the same time, the cells without treatment served as the control.

### 2.13 3‐[4,5‐dimethylthiazol‐2‐yl]‐2,5‐diphenyltetrazolium bromide (MTT) assay

Following H_2_O_2_ treatment for 12 hours, the H9c2 cells were centrifuged at 503.1 *g* for 5 minutes, and the culture medium in the well was renewed with MTT solution (1 mg/mL) that had been prepared with fresh culture medium in the dark. The culture plate was cultured in a cell incubator for 3 hours. The MTT solution was discarded after centrifugation at 1509.3 *g* for 6 min, and 100 μL DMSO was added into each well. The optical density (OD) values at 450 nm were measured using a microplate. The cell survival rate was calculated using the following formula: cell survival rate = 100% × (average OD value of treatment group/average OD value of control group).

### 2.14 Flow cytometry

Apoptosis was assessed by Annexin V‐fluorescein isothiocyanate (FITC)/propidium iodide (PI) via the double‐staining method. Briefly, the H9c2 cells were treated with H_2_O_2_ for 12 hours, rinsed twice using 4°C precooled PBS, trypsinized and placed into a 15‐mL centrifuge tube for centrifugation at 800 g. Following supernatant removal, the precipitates were rinsed twice with PBS, after which the cells were re‐suspended in 500‐μL binding buffer in accordance with the Annexin V‐FITC apoptosis detection kit instructions (BD Biosciences). Afterwards, 5 μL FITC and 5 μL PI were added into the buffer, followed by sufficient mixing. After incubation for 15 minutes, apoptosis was detected using a flow cytometer (BD).

### 2.15 Reactive oxygen species (ROS) determination

The H9c2 cells or myocardial tissues that had been treated with H_2_O_2_ were rinsed twice with 4°C precooled PBS. Following the instructions of 2′,7′‐dichlorodihydrofluorescein diacetate (DCFDA)/H2DCFDA‐Cellular ROS Assay Kit (Abcam), the cells were incubated with 10 mmol/L DCFDA at 4°C for 45 minutes. The fluorescence change was recorded using a fluorophotometer at a maximum excitation wavelength of 495 nm and maximum emission wavelength of 527 nm. The ROS level in H9c2 cells or myocardial tissues was calculated based on the fluorescence intensity of DCF.

### 2.16 Enzyme linked immunosorbent assay (ELISA)

The H9c2 cells were treated with H_2_O_2_, rinsed twice with pre‐cooled PBS and trypsinized. The cell precipitates were collected and homogenized at 4°C with precooled PBS, and subsequently centrifuged at 800 g in order to obtain the supernatant. The protein concentration of the supernatant was determined using the BCA (Pierce) method, with 30 μg protein prepared for each sample group for subsequent determination. NBT/enzyme working solution was added to the samples for incubation at 37°C for 30 minutes in accordance with the instructions provided by Total Superoxide Dismutase Assay Kit with nitroblue tetrazolium (NBT; Beyotime). The OD was measured at 560 nm, and the activity of superoxide dismutase (SOD) in H9c2 cells was measured based on the OD value. Catalase detection buffer was added to the samples, followed by incubation at 25°C for 5 minutes based on the specifications of the Catalase (CAT) Assay Kit (Beyotime). The OD values were measured at 520 nm, based on which the CAT activity in H9c2 cells was calculated.

Next, 3 mL of peripheral venous blood was collected from rats and placed into EDTA tubes, with the plasma component subsequently separated via centrifugation at 4°C and 3000 g for 10 minutes within 2 hours. The levels of brain natriuretic peptide (BNP) and creatine kinase (CKMB) were detected in accordance with the instructions of Rat BNP 45 ELISA Kit (Abcam) and Rat CKMB ELISA Kit (Elabscience). The OD values were measured at 450 nm wavelength using a microplate. Expression of SOD and CAT in serum was determined by ELISA.

### 2.17 Dual‐luciferase reporter gene assay

The predicted mRNA 3’‐UTR fragment containing the miR‐454‐binding site and the mutation fragment of NEDD4‐2 were subsequently inserted into the Pmir‐GLO Dual‐Luciferase miRNA Target Expression Vectors (Promega Corporation) to generate the reporter plasmids NEDD4‐2‐wild type (WT) and NEDD4‐2‐mutant type (MUT). mimic NC and miR‐454 mimic were co‐transfected with NEDD4‐2 Luciferase Reporter plasmids into 293T cells (Oulu Biotechnology), respectively, to ascertain whether miR‐454 could combine with the mRNA 3’‐UTR of NEDD4‐2. Next, 293T cells were cultured with DMEM (Gibco) containing 10% FBS (Gibco), 100 μg/mL streptomycin and 100 U/mL penicillin in an incubator (Thermo Fisher Scientific Inc) at 37°C with 5% CO_2_. The cells were then collected and lysed 48 hours post‐transfection. Dual‐luciferase reporter gene assay was performed in an analytical system (Promega) using a luciferase detection kit (K801‐200, BioVision, Palo Alto). With renilla luciferase as an internal reference, the activation degree of the target reporter gene was expressed as the ratio between firefly luciferase and renilla luciferase.

### 2.18 RNA immunoprecipitation (RIP) assay

RIP assay was performed using the Magna RIP Kit (Millipore) as per the manufacturer's instructions. Briefly, the H9c2 cells were lysed in RIP lysis buffer, then 100 μL of whole cell extract was incubated with RIP buffer containing magnetic beads conjugated with human antiAgo2 antibody, negative control normal mouse IgG (Millipore). Next, to digest the protein, the samples were incubated with proteinase K with shaking, after which the immunoprecipitated RNA was isolated with the use of phenol‐chloroform‐isoamyl alcohol (Sigma Aldrich) and finally the RNA pellet was visualized using sodium acetate/ethanol‐precipitated together with RNA‐grade glycogen (Thermo Scientific) and resuspended in RNase‐free water. Finally, the levels of miR‐454 and NEDD4‐2 mRNA in the precipitates were detected by RT‐qPCR.

### 2.19 Co‐immunoprecipitation (Co‐IP)

The cells were harvested 48 hours post‐transfection and lysed with cell lysis buffer containing protease inhibitor on ice for 30 minutes. One part of the lysate (30 μg of total protein) was used as the input or incubated with 2 μg of NEDD4‐2 antibody (anti‐rabbit, Sigma), while 2 μg of IgG (anti‐rabbit, Sigma) was added to the other part as the NC. Next, 10 μL protein A agarose beads were washed with lysis buffer three times and centrifuged at 3000 rpm for 3 minutes. The beads were subsequently incubated with the cell lysate that had been incubated overnight with the antibody for 3 hours at 4°C, to couple the antibody. Following immunoprecipitation reaction, the agarose beads were centrifuged to the bottom of the tube via centrifugation at 4°C and 3000 rpm for 3 minutes. Following protein concentration determination, 15 μL of 2 × sodium dodecyl sulphate buffer was added to the protein, followed by boiling for 5 minutes, followed by Western blot assay.

### 2.20 Protein half‐life determination

After 24 hours of transfection, the cells in the 10‐cm cell‐culture dish were separately placed into six 6‐cm cell‐culture dishes. After 24 hours of cell growth, the protein synthesis inhibitor cycloheximide (CHX) was added to the cells at a final concentration of 40 μg/mL, after which the cells were collected at various time points (0, 2, 4, 6, 8, 10 hours). The expression of TrkA (anti‐rabbit, Sigma) was measured by Western blot assay.

### 2.21 Analysis of protein ubiquitination

Next, 20 μmol/L MG‐132, a protease inhibitor, was added to the H9c2 cells following 48 hours of plasmid transfection. After 6 hours of treatment with MG‐132, the cells were lysed using a lysis buffer that had been supplemented with protease inhibitor for 30 minutes on ice. The protein immunoprecipitation complex was obtained using 2‐μg TrkA antibody (anti‐rabbit, Sigma). The expression of TrkA (anti‐rabbit, 1:100, Sigma) and ubiquitin (anti‐rabbit, 1:10 000, Abcam) was analysed by Western blot assay.

### 2.22 Statistical analysis

SPSS version 21.0 (IBM) was utilized to evaluate data in this study. All data are summarized by the mean ± standard deviation, with *P* < .05 as a level of statistically significance. Unpaired *t*‐test was conducted for data comparison between two groups, while one‐way analysis of variance (ANOVA) was applied for data comparison among multiples in combination with a Tukey's posthoc test. Two‐way ANOVA was conducted for data comparison at different time points, while a Pearson's correlation analysis was performed to analyse the correlation between indicators.

## 3 RESULTS

### 3.1 miR‐454 is downregulated in HF and negatively correlated with the grade of HF

**FIGURE 1:**
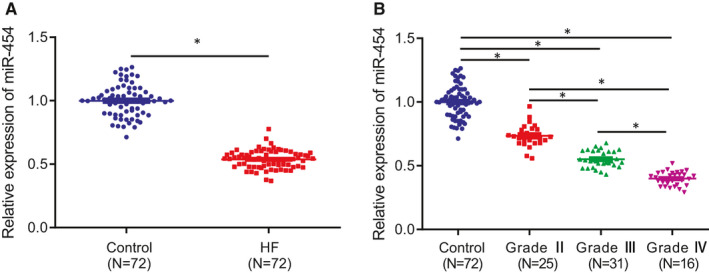
miR‐454 is downregulated in HF and negatively correlated with the grade of HF. A, The expression of miR‐454 in peripheral blood of HF patients with AMI complicated with HF and healthy individuals determined by RT‐qPCR; n = 72; *P < .05. B, miR‐454 expression in peripheral blood of healthy individuals and patients with different grades of HF determined by RT‐qPCR; *P < .05. Measurement data are displayed as mean ± standard deviation. Independent sample *t*‐tests were used for comparing data between the two groups, and one‐way ANOVA for comparison of data among multiple groups

RT‐qPCR was performed to determine the expression of miR‐454 in peripheral blood of 72 patients with AMI complicated with HF and 72 healthy individuals (control). In comparison with the peripheral blood of healthy individuals, the expression of miR‐454 was notably downregulated in peripheral blood of patients with acute HF (Figure 1A). The 72 patients with acute HF were classified into grade II, III and IV based on the Killip classification. Our results also revealed that the expression of miR‐454 was negatively correlated with the different grades of HF (*P* < .05; Figure 1B).

### 3.2 Overexpression of miR‐454 impairs apoptosis of H9c2 cells

**FIGURE 2:**
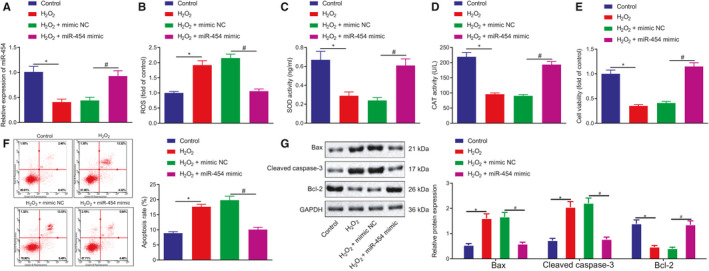
Overexpression of miR‐454 inhibits H9c2 cell apoptosis. H9c2 cells were non‐treated or treated with H_2_O_2_, H_2_O_2_ + mimic NC or H_2_O_2_ + miR‐454 mimic; H_2_O_2_ was added for another 12 h after plasmids transfection for 36 h. A, The expression of miR‐454 in H9c2 cells determined by RT‐qPCR; */^#^ *P* < .05. B, ROS levels in H9c2 cells; */^#^ *P* < .05. C, The activities of SOD in H9c2 cells determined by ELISA; */^#^ *P* < .05. D, The activities of CAT in H9c2 cells determined by ELISA; */^#^ *P* < .05. E, The survival rates of H9c2 cells examined by MTT assay; */^#^ *P* < .05. F, The apoptotic rates of H9c2 cells as examined by flow cytometry; */^#^ *P* < .05. G, The protein expression of Bax, cleaved caspase‐3 and Bcl‐2 in H9c2 cells determined by Western blot assay; */^#^ *P* < .05. Measurement data are displayed as mean ± standard deviation. One‐way ANOVA was performed for comparison of data among multiple groups. Each cell experiment was repeated three times

Next, to elucidate the effect of miR‐454 on myocardial apoptosis during HF, we overexpressed miR‐454 in H9c2 cells which were induced by H_2_O_2_ to establish an oxidative stress model for HF in vitro simulation purposes. The results of RT‐qPCR revealed that the expression of miR‐454 in H9c2 cells treated with H_2_O_2_ was markedly lower than that in control cells, while increased levels were detected following miR‐454 mimic transfection, which validated the successful transfection efficiency of miR‐454 mimic (Figure 2A). The ROS level in H9c2 cells was elevated following induction with H_2_O_2_, while a marked decrease in the ROS level was detected after transfection with miR‐454 mimic (Figure 2B). After H_2_O_2_ induction, the SOD and CAT activities in the H9c2 cells were both markedly diminished, the effects of which were reversed following transfection with miR‐454 mimic (Figure 2C, D). Additionally, H_2_O_2_‐induced oxidative stress injury led to a decrease in survival rate accompanied by an elevated rate of apoptosis in the H9c2 cells, elevated expression of pro‐apoptotic proteins Bax and cleaved caspase‐3, along with decreased expression of anti‐apoptotic protein Bcl‐2; whereas, miR‐454 overexpression led to a reduction in the aforementioned changes and upregulated Bcl‐2 expression (Figure 2E‐G). Taken together, miR‐454 expression is decreased in the oxidative stress model in vitro and overexpression of miR‐454 inhibits the oxidative stress‐induced apoptosis of H9c2 cells.

### 3.3 **miR**‐**454 targets NEDD4‐2 in H9c2 cells**

**FIGURE 3:**
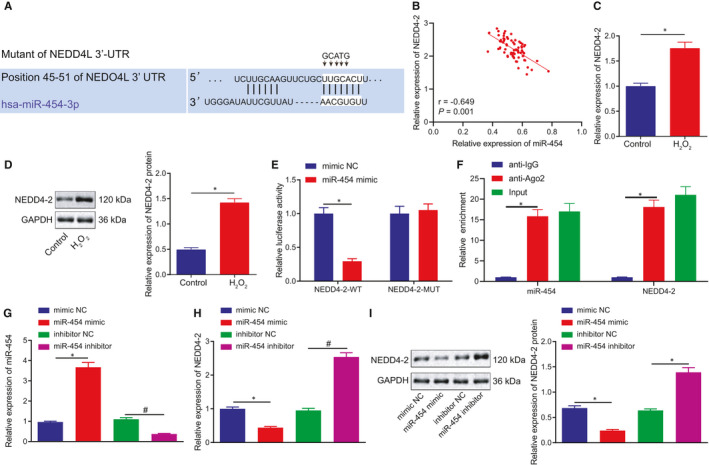
miR‐454 negatively modulates NEDD4‐2 expression in H9c2 cells. A, Bioinformatics analysis of binding site between miR‐454 and NEDD4‐2. B, Correlation analysis of the expression of miR‐454 and NEDD4‐2 in 72 patients with HF determined with immunohistochemistry. C, The mRNA expression of NEDD4‐2 in non‐treated H9c2 cells or H9c2 cells treated with H_2_O_2_ for 12 h as measured by RT‐qPCR; *P < .05. D, The protein expression of NEDD4‐2 in non‐treated H9c2 cells or H9c2 cells treated with H_2_O_2_ as measured by Western blot assay; *P < .05. E, Dual‐luciferase reporter gene assay examining whether miR‐454 binds to NEDD4‐2 mRNA; *P < .05. F, Cellular lysates from H9c2 cells were used for RIP with Ago2 antibody. miR‐454 and NEDD4‐2 mRNA were detected using RT‐qPCR. In panels G‐I, H9c2 cells transfected with mimic NC, miR‐454 mimic, inhibitor NC or miR‐454 inhibitor. G, The expression of miR‐454 in H9c2 cells determined by RT‐qPCR 48 h after transfection; */^#^ *P* < .05. H, The mRNA expression of NEDD4‐2 in H9c2 cells determined by RT‐qPCR 48 h after transfection; */^#^ *P* < .05. I, The protein expression of NEDD4‐2 in H9c2 cells determined by Western blot assay 48 h after transfection; */^#^ *P* < .05. Measurement data are displayed as mean ± standard deviation. Independent sample *t*‐tests were used for comparing data between the two groups, and one‐way ANOVA for comparison of data among multiple groups. Pearson's correlation analysis was performed for observation of the correlation between miR‐454 and NEDD4‐2 mRNA expression. Each cell experiment was repeated three times

In an attempt to further identify the mechanism by which miR‐454 influences HF, TargetScan was employed to predict the downstream targets of miR‐454. Bioinformatics analysis provided data identifying the miR‐454 binding sites in NEDD4‐2 (Figure 3A). Hence, we set out to evaluate whether miR‐454 could suppress NEDD4‐2. Subsequently, we assessed the expression of miR‐454 and NEDD4‐2 in patients with HF using Immunohistochemistry, which identified negative correlation between the expression of NEDD4‐2 and miR‐454 (Figure 3B). Compared with the control cells, H9c2 cells treated with H_2_O_2_ displayed notably elevated NEDD4‐2 at both mRNA and protein levels (Figure 3C, D). The results of dual‐luciferase reporter assay demonstrated that the luciferase activity was notably decreased when the cells were co‐transfected with NEDD4‐2‐WT and miR‐454 mimic rather than NEDD4‐2‐MUT and miR‐454 mimic (Figure 3E). Next, to further explore whether both miR‐454 and NEDD4‐2 exist in RISC complex, we performed RIP experiments on H9c2 cell extracts using antibodies against Ago2, a key component of RISC complex. As expected, miR‐454 and NEDD4‐2 were enriched in Ago2 pellets to control IgG immunoprecipitation (Figure 3F). RT‐qPCR and Western blot assay results revealed that the mRNA and protein expression of NEDD4‐2 in H9c2 cells were reduced following treatment with miR‐454 mimic, which was elevated after treatment with miR‐454 inhibitor (Figure 3G‐I). In summary, the aforementioned findings suggest that miR‐454 targets NEDD4‐2 to inhibit its expression in H9c2 cells.

### 3.4 miR‐454 targets NEDD4‐2 to repress H9c2 cell apoptosis and injury

**FIGURE 4:**
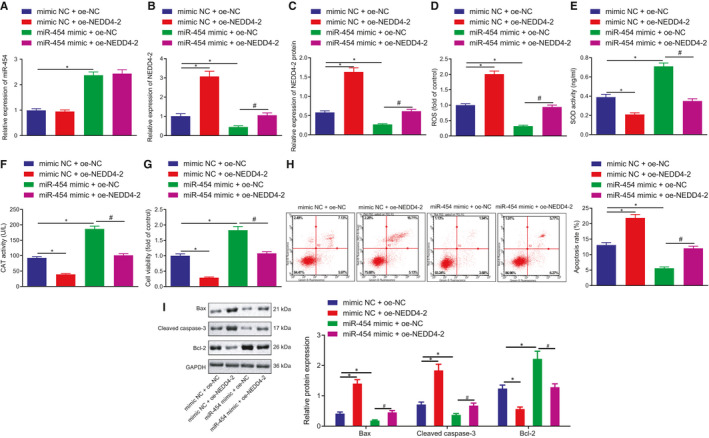
miR‐454‐dependent downregulation of NEDD4‐2 attenuates H9c2 cell apoptosis and injury. H9c2 cells co‐transfected with mimic NC + oe‐NC, mimic NC + oe‐NEDD4‐2, miR‐454 mimic + oe‐NC or miR‐454 mimic + oe‐NEDD4‐2 for 48 h. A, The expression of miR‐454 in H9c2 cells determined by RT‐qPCR; */^#^ *P* < .05. B, C The mRNA and protein expression of NEDD4‐2 in H9c2 cells determined by RT‐qPCR and Western blot assay; */^#^ *P* < .05. D, ROS levels in H9c2 cells; */^#^ *P* < .05. E, The activities of SOD in H9c2 cells as examined by ELISA; */^#^ *P* < .05. F, The activities of CAT in H9c2 cells as measured by ELISA; */^#^ *P* < .05. G, The survival rates of H9c2 cells as examined by MTT assay; */^#^ *P* < .05. H, The apoptotic rates of H9c2 cells examined by flow cytometry; */^#^ *P* < .05. I, The protein expression of Bax, cleaved caspase‐3 and Bcl‐2 in H9c2 cells determined by Western blot assay; */^#^ *P* < .05. Measurement data are displayed as mean ± standard deviation. One‐way ANOVA was performed for comparison of data among multiple groups. Each cell experiment was repeated three times

The rescue experiments were subsequently performed to ascertain whether miR‐454 affects H9c2 cell apoptosis and injury by targeting NEDD4‐2. The RT‐qPCR results revealed that transfection with oe‐NEDD4‐2 contributed to an increase in mRNA and protein expression of NEDD4‐2 in H_2_O_2_‐exposed H9c2 cells, while miR‐454 mimic successfully upregulated the expression of miR‐454 which was accompanied by a reduction in the expression of NEDD4‐2. Moreover, oe‐NEDD4‐2 rescued the expression of NEDD4‐2 that was reduced in the setting of miR‐454 overexpression, which validated the successful transduction efficiency of oe‐NEDD4‐2 (Figure 4A‐C). Additionally, NEDD4‐2 overexpression led to increased ROS level and reduced SOD and CAT activities, while an opposite trend of results was detected in response to miR‐454 gain‐of‐function. Notably, NEDD4‐2 was found to neutralize the effects triggered by miR‐454 overexpression on the aforementioned indicators (Figure 4D‐F). Upon NEDD4‐2 overexpression, the cell survival rate was decreased and the apoptotic rate was increased, accompanied with elevated Bax and cleaved caspase‐3 protein expression and reduced Bcl‐2 protein expression. On the contrary, enhanced cell viability and reduced cell apoptosis along with reduction in Bax and cleaved caspase‐3 protein expression and increase in Bcl‐2 protein expression were detected in the presence of miR‐454, all of which could be reversed following the restoration of NEDD4‐2 (Figure 4G‐I). Altogether, the results highlight that inhibition of NEDD4‐2 is responsible for the anti‐apoptotic and protective effects of miR‐454 on H9c2 cells.

### 3.5 NEDD4‐2 induces ubiquitination and degradation of TrkA protein in H9c2 cells

**FIGURE 5:**
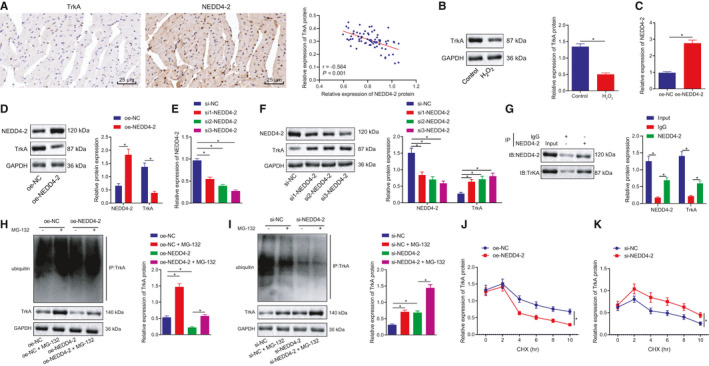
NEDD4‐2 specifically binds to TrkA receptor protein and stimulates the ubiquitination and degradation of TrkA protein in H9c2 cells. A, Correlation analysis between the expression of NEDD4‐2 and miR‐454 determined with immunohistochemistry. B, The protein expression of TrkA in control H9c2 cells or H9c2 cells treated with H_2_O_2_ for 12 h as measured by Western blot assay; *P < .05. C, The mRNA expression of NEDD4‐2 in H9c2 cells treated with oe‐NC or oe‐NEDD4‐2 determined by RT‐qPCR 48 h after transfection; *P < .05. D, The protein expression of NEDD4‐2 in H9c2 cells treated with oe‐NC or oe‐NEDD4‐2 determined by Western blot assay 48 h after transfection; *P < .05. E, The mRNA expression of NEDD4‐2 in H9c2 cells treated with si‐NC, si1‐NEDD4‐2, si2‐NEDD4‐2 and si3‐NEDD4‐2 determined by RT‐qPCR 48 h after transfection; *P < .05. F, The protein expression of NEDD4‐2 and TrkA in H9c2 cells treated with si‐NC, si1‐NEDD4‐2, si2‐NEDD4‐2 and si3‐NEDD4‐2 determined by Western blot assay 48 h after transfection; *P < .05. G, The binding of NEDD4‐2 to TrkA in H9c2 cells determined by Co‐IP assay. H, The ubiquitination level of TrkA protein in H9c2 cells upon NEDD4 overexpression 48 h after transfection. I, The ubiquitination level of TrkA protein in H9c2 cells upon NEDD4 silencing 48 h after transfection. J, The TrkA protein halflife determination in H9c2 cells upon NEDD4 overexpression 48 h after transfection. K, The TrkA protein half‐life determination in H9c2 cells upon NEDD4 silencing 48 h after transfection. Measurement data are displayed as mean ± standard deviation. Independent sample *t*‐tests were used for comparing data between the two groups, and one‐way ANOVA for comparison of data among multiple groups. Pearson's correlation analysis was performed for observation of the correlation between TrkA and NEDD4‐2 protein expression. Each cell experiment was repeated three times

Existing literature has suggested that NEDD4‐2 ubiquitinated TrkA and TrkA are aberrantly downregulated in HF tissues.^15^, ^17^, ^21^ We subsequently set out to investigate whether NEDD4‐2 plays a role in H9c2 cells by regulating TrkA expression. As reflected by immunohistochemistry, a negative correlation was observed between the expression of NEDD4‐2 and miR‐454 (Figure 5A) As expected, H_2_O_2_ stimulation also resulted in a marked decline in the protein expression of TrkA in H9c2 cells (Figure 5B). NEDD4‐2 overexpression in the H_2_O_2_‐exposed H9c2 cells resulted in increased protein expression of NEDD4‐2 and decreased protein expression of TrkA (Figure 5C, D). Additionally, three siRNAs were designed to silence NEDD4‐2 in H_2_O_2_‐exposed H9c2 cells, which was subsequently confirmed by the reductions detected in the mRNA and protein expression of NEDD4‐2, as reflected by RT‐qPCR and Western blot assay. The silencing triggered a reduction in NEDD4‐2 expression and elevation in TrkA protein expression. The si3‐NEDD4‐2 with the highest degree of interference efficiency was employed for subsequent experiments (Figure 5E, F). Based on the aforementioned findings, we speculate that NEDD4‐2, as an E3 enzyme, may ubiquitinate and promote TrkA degradation. In the Co‐IP experiment, evidence was obtained suggesting that the NEDD4‐2 protein bound to TrkA protein in H9c2 cells (Figure 5G). Following NEDD4‐2 overexpression, the ubiquitination level of TrkA protein was markedly increased, while the expression of TrkA protein displayed a decrease. After treatment with MG‐132, an inhibitor for ubiquitination degradation, the ubiquitination level of TrkA protein decreased along with increased levels of TrkA protein expression in the presence of NEDD4‐2. Upon NEDD4‐2 knockdown, the ubiquitination level of TrkA protein was reduced while the protein expression of TrkA was notably increased, with the effects of NEDD4‐2 knockdown observed to be further enhanced after co‐treatment with MG‐132 (Figure 5H, I). Next, the halflife of TrkA protein decreased upon NEDD4‐2 overexpression and increased upon NEDD4‐2 knockdown (Figure 5J, K). The aforementioned results suggest that NEDD4‐2 specifically binds to TrkA receptor protein, thereby stimulating the ubiquitination and degradation of TrkA protein in H9c2 cells.

### 3.6 miR‐454 upregulates TrkA to attenuate H9c2 cell apoptosis and injury by targeting NEDD4‐2

**FIGURE 6:**
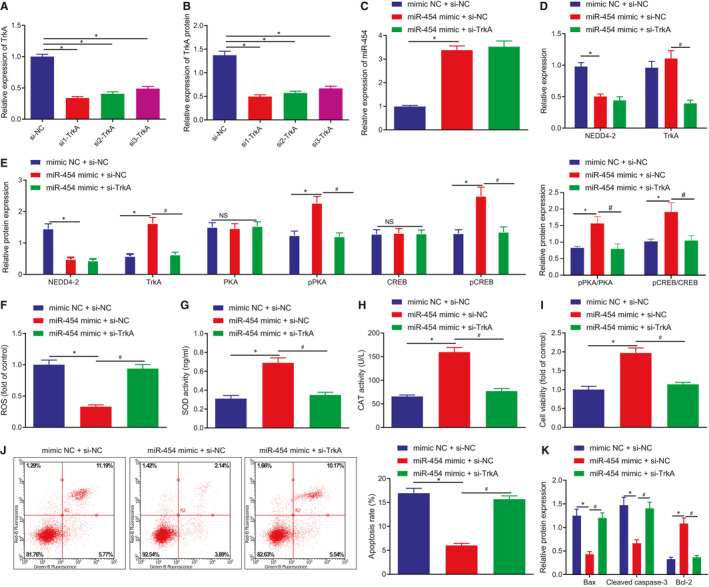
miR‐454 upregulates TrkA to inhibit H9c2 cell apoptosis and injury by targeting NEDD4‐2. A, The mRNA of TrkA in H9c2 cells treated with si‐NC, si1‐TrkA, si2‐TrkA or si3‐TrkA determined by RT‐qPCR 48 h after transfection; */^#^ *P* < .05. B, The protein expression of TrkA in H9c2 cells treated with si‐NC, si1‐TrkA, si2‐TrkA or si3‐TrkA determined by Western blot assay 48 h after transfection; */^#^ *P* < .05. In panels C–K, H9c2 cells were treated with mimic NC + si‐NC, miR‐454 mimic + si‐NC or miR‐454 mimic + si‐TrkA for 48 h. C, The expression of miR‐454 in H9c2 cells determined by RT‐qPCR; */^#^ *P* < .05. D, The mRNA expression of NEDD4‐2 and TrkA in H9c2 cells determined by RT‐qPCR; */^#^ *P* < .05. E, The protein expression of NEDD4‐2 and TrkA and the extents of PKA and CREB phosphorylation in H9c2 cells determined by Western blot assay; */^#^ *P* < .05. F, ROS levels in H9c2 cells; */^#^ *P* < .05. G, The activities of SOD in H9c2 cells as examined by ELISA; */^#^ *P* < .05. H, The activities of CAT in H9c2 cells as measured by ELISA; */^#^ *P* < .05. I, The survival rates of H9c2 cells as examined by MTT assay; */^#^ *P* < .05. (J) The apoptotic rates of H9c2 cells as examined by flow cytometry; */^#^ *P* < .05. K, The protein expression of Bax, cleaved caspase‐3 and Bcl‐2 in H9c2 cells determined by Western blot assay; */^#^ *P* < .05. Measurement data are displayed as mean ± standard deviation. One‐way ANOVA was performed for comparison of data among multiple groups. Each cell experiment was repeated three times

Three siRNAs were designed to silence TrkA in H9c2 cells. RT‐qPCR and Western blot assay results provided verification of the knockdown efficiency of those siRNAs with reductions detected in both mRNA and protein expression of TrkA, among which si1‐TrkA achieved the highest interference efficiency, so si1‐TrkA was chosen for subsequent experiments (Figure 6A, B). The H9c2 cells exposed to H_2_O_2_ were co‐transfected with mimic‐NC/miR‐454 mimic and si‐NC/si1‐TrkA to analyse whether TrkA was required for the protective function of miR‐454 on H_2_O_2_‐induced cell damage. As expected, miR‐454 gain‐of‐function contributed to a decrease in the mRNA and protein expression of NEDD4‐2, but increased in protein expression of TrkA and the extents of phosphorylation of its downstream CAMP pathway markers PKA and CREB. Reversely, TrkA knockdown in the presence of miR‐454 reduced the extents of PKA and CREB phosphorylation (Figure 6C‐E). Furthermore, the diminished ROS level and increased SOD and CAT activities induced by miR‐454 were reversed by TrkA knockdown in H_2_O_2_‐exposed H9c2 cells (Figure 6F‐H). Meanwhile, the enhancement of cell survival and suppression of apoptosis caused by miR‐454 were neutralized by TrkA silencing, a finding of which was supported by the changes in pro‐apoptotic and anti‐apoptotic proteins in H_2_O_2_‐exposed H9c2 cells (Figure 6I‐K). Taken together, miR‐454 reduces oxidative stress‐evoked apoptosis and injury of H9c2 cells by upregulating TrkA through inhibition of NEDD4‐2.

### 3.7 miR‐454 overexpression protects H9c2 cells from apoptosis by activating the cAMP pathway

**FIGURE 7:**
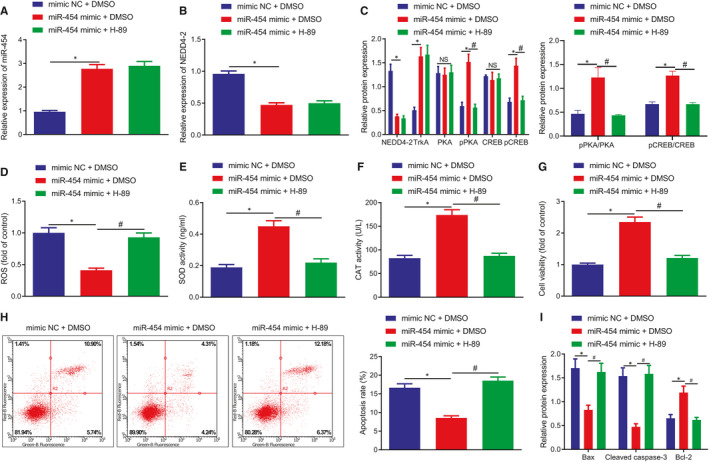
miR‐454 inhibits cardiomyocyte apoptosis and injury by activating the cAMP pathway through NEDD4‐2/TrkA axis. H9c2 cells were treated with mimic NC + DMSO, miR‐454 mimic + DMSO or miR‐454 mimic + H‐89 for 48 h. A, The expression of miR‐454 in H9c2 cells determined by RT‐qPCR. B, The mRNA expression of NEDD4‐2 in H9c2 cells determined by RT‐qPCR; */^#^ *P* < .05. C, The protein expression of NEDD4‐2 and TrkA and the extents of PKA and CREB phosphorylation in H9c2 cells determined by Western blot assay; */^#^ *P* < .05. D, ROS levels in H9c2 cells; */^#^ *P* < .05. E, The activities of SOD in H9c2 cells examined by ELISA; */^#^ *P* < .05. F, The activities of CAT in H9c2 cells measured by ELISA; */^#^ *P* < .05. G, The survival rates of H9c2 cells examined by MTT assay; */^#^ *P* < .05. H, The apoptotic rates of H9c2 cells as examined by flow cytometry; */^#^ *P* < .05. I, The protein expression of Bax, cleaved caspase‐3 and Bcl‐2 in H9c2 cells determined by Western blot assay; */^#^ *P* < .05. Measurement data are displayed as mean ± standard deviation. One‐way ANOVA was performed for comparison of data among multiple groups. Each cell experiment was repeated three times

Next, to ascertain whether miR‐454 could suppress the apoptosis and injury of H9c2 cells through the NEDD4‐2/TrkA/cAMP axis, miR‐454 was overexpressed in H_2_O_2_‐exposed H9c2 cells, which were simultaneously treated with H‐89, the inhibitor of cAMP pathway. miR‐454 gain‐of‐function led to downregulated NEDD4‐2 expression, elevated TrkA protein expression and enhanced phosphorylation of PKA and CREB. H‐89 treatment led to a decrease in the extents of PKA and CREB phosphorylation in the presence of miR‐454 (Figure 7A‐C). We then speculated that miR‐454 protected the H9c2 cells through NEDD4‐2/TrkA/cAMP axis. As expected, reduced ROS level and increased SOD and CAT activities caused by miR‐454 overexpression were neutralized by treatment with H‐89 (Figure 7D‐F). Consistently, enhanced cell viability and suppressed apoptosis, in addition to reduced Bax and cleaved caspase‐3 protein expression and elevated Bcl‐2 protein expression induced by miR‐454 were reversed by treatment with H‐89 in the H_2_O_2_‐exposed H9c2 cells (Figure 7G‐I). The aforementioned results suggest that miR‐454 elevation activates the cAMP pathway to protect against oxidative stress‐evoked H9c2 cells apoptosis and injury.

### 3.8 miR‐454 activates cAMP pathway through NEDD4‐2/TrkA axis to delay HF in vivo

**FIGURE 8:**
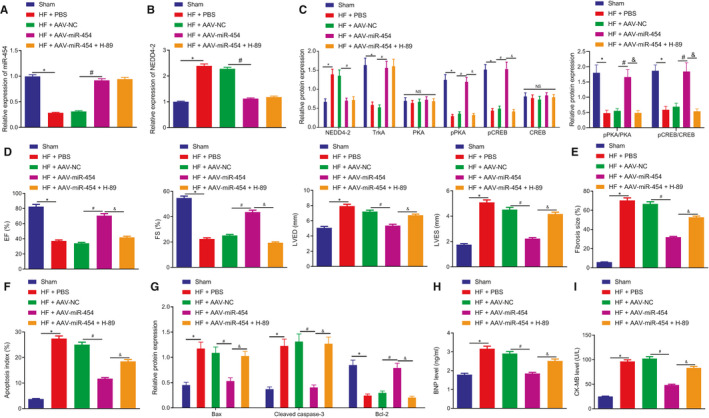
miR‐454 activates cAMP pathway through NEDD4‐2/TrkA axis to delay HF in vivo. The rats were grouped into sham‐operated rats, and HF rats treated with PBS, AAV‐NC, AAV‐miR‐454, and AAV‐miR‐454 + H‐89, respectively. A, The expression of miR‐454 in the heart tissues determined by RT‐qPCR; n = 12; */^#^/&*P* <.05. B, The expression of NEDD4‐2 in the heart tissues determined by RT‐qPCR; n = 12; */^#^/&*P* < .05. C, The protein expression of NEDD4‐2 and TrkA and the extents of PKA and CREB phosphorylation in the heart tissues as measured by Western blot assay; n = 12; */^#^/&*P* < .05. D, The cardiac functions as examined by colour Doppler ultrasound (EF, FS, LVED, LVES and heart rate); n = 12; */^#^/&*P* < .05. E, The degree of myocardial fibrosis as examined by Masson's Trichrome staining; n = 12. F, The apoptotic rate in myocardial tissues as examined by TUNEL staining; n = 12; */^#^/&*P* < .05. G, The protein expression of Bax, cleaved caspase‐3 and Bcl‐2 in heart tissues as examined by Western blot assay; n = 12; */^#^/&*P* < .05. H, The expression of BNP in plasma as examined by ELISA; n = 12; */^#^ &*P* < .05. I, The expression of CK‐MB in plasma as examined by ELISA; n = 12; */^#^ &*P* < .05. Measurement data are displayed as mean ± standard deviation. One‐way ANOVA was performed for comparison of data among multiple groups

The emphasis was subsequently shifted to the involvement of miR‐454/NEDD4‐2/TrkA/cAMP axis in the apoptosis and injury of H9c2 cells, as well as the cardiac function following HF. A HF rat model was constructed (Figure S1A), after which AAV‐miR‐454 was injected in situ, and H‐89, an inhibitor of cAMP pathway, was intraperitoneally injected into the rats. When compared to the sham‐operated rats, the HF rat models exhibited a decrease in the expression of miR‐454 and increase in mRNA and protein expression of NEDD4‐2 in the heart tissues and serum, accompanied with decreased TrkA protein expression and the extents of PKA and CREB phosphorylation. AAV‐miR‐454 treatment was found to contribute to an increase in the expression of miR‐454 and decreased mRNA and protein expression of NEDD4‐2 in the heart tissues and serum of rats rendered with HF, along with increased TrkA protein expression and the extents of PKA and CREB phosphorylation. H‐89 treatment abolished the PKA and CREB phosphorylation enhanced by AAV‐miR‐454 (Figure 8A‐C; Figure S1B). Moreover, echocardiography, HE staining and Masson's Trichrome staining methods were performed to evaluate cardiac function, myocardial damage and fibrosis. The HF rats exhibited heart function impairment supported by reduction in EF and FS, and increase in LVED, LVES and heart rate. AAV‐miR‐454 alleviated HF‐induced cardiac dysfunction, myocardial interstitial collagen and myocardial fibrosis accompanied with increased EF and FS, but reduced LVED, LVES and heart rate. H‐89 aggravated the HF‐induced cardiac dysfunction and the myocardial damage and fibrosis in HF that could be alleviated by AAV‐miR‐454, accompanied with reduced EF and FS, but increased LVED, LVES and heart rate (Figure 8D, E, Figure S2A‐C). Furthermore, the TUNEL and Western blot assay results demonstrated that compared with sham‐operated rats, the HF rats had increased apoptotic rates, elevated expression of Bax and cleaved caspase‐3 and decreased Bcl‐2 expression in the myocardial tissues. AAV‐miR‐454 led to a decrease in the rate of apoptosis, reduced expression of Bax and cleaved caspase‐3 and increased Bcl‐2 expression in the myocardial tissues of rats rendered with HF, whereas all of which could be reversed by treatment with H‐89 (Figure 8F, G, Figure S2D). The ROS level as well as the CAT and SOD activities in each group were detected (Figure S1C‐E). The data obtained suggested that compared with the sham‐operated rats, the levels of ROS increased (*P* < .05), and the SOD and CAT activities were both decreased (*P* < .05) in the PBS‐injected rats after modelling. There were no significant changes in ROS level, SOD and CAT activity in the modelled rats following injection with AAV‐NC and PBS. Besides, in comparison with treatment with AAV‐NC following model establishment, injection with AAV‐miR‐454 to overexpress miR‐454 after modelling resulted in decreased ROS level (*P* < .05) and increased SOD and CAT activities (*P* < .05). Moreover, AAV‐miR‐454 + H‐89 injection was performed after modelling to overexpress and inhibit the cAMP pathway simultaneously, which was observed to result in increased ROS level and restrained SOD and CAT activities (*P* < .05) in contrast to the AAV‐miR‐454 injection after modelling. Based on the ELISA results, compared with that in plasma of sham‐operated rats, the expression of HF markers BNP and CK‐MB exhibited a marked increase in the plasma of the HF rat models. Treatment with AAV‐miR‐454 notably decreased the expression of BNP and CK‐MB, which could be neutralized by H‐89 treatment (Figure 8H, I). Taken together, miR‐454 stimulates the cAMP pathway activation through NEDD4‐2/TrkA axis in vivo to delay HF.

## 4 DISCUSSION

HF represents a complex disorder that is responsible for a huge societal burden in regard to both cost and overall fatality.^22^ Accumulating evidence continues to implicate miRs in the progression of various heart diseases including HF.^23^ During the current study, we set out to elucidate the function of miR‐454 in HF and demonstrated its cardioprotective effects with the involvement of NEDD4‐2‐regulated TrkA ubiquitination and cAMP pathway.

Our initial observations revealed that the expression of miR‐454 was downregulated in the peripheral blood of patients with HF as well as in H_2_O_2_‐treated H9c2 cells. As a form of cardiac myoblasts, H9c2 cells have been widely employed for the study of cardiomyopathy, which have been reported to exhibit almost the same proliferative response as primary cardiomyocytes.^24^ Existing literature has highlighted the importance of H9c2 cells as a model for in vitro cardiac hypertrophy studies and supports the current work of human cardiomyocyte cell lines in prospective molecular studies of cardiac development and disease^25^, ^26^, ^27^ Hence, during the current study, we selected H9c2 as our model to simulate cardiomyocytes in vitro. Consistent with the findings of our study, circulating miR‐454 has been previously reported to be downregulated in patients with diastolic dysfunction.^8^ The overexpression of miR‐454 has been speculated to be a promising potential therapeutic target for the alleviation of acute lung injury.^28^ We have uncovered that in the event of treatment with H_2_O_2_, the expression level of miR‐454 decreases, which in turn triggers an increase in ROS levels, decreased SOD and CAT levels, decreased cell viability, as well as an increased rate of cell apoptosis and apoptosis‐related proteins (Figure 2). Apart from the hepato‐protective role of miR‐454,^29^ our study further highlighted the cardioprotective role of miR‐454 in H9c2 cells by antagonizing apoptosis. This function may potentially be achieved *via* the downregulation of NEDD4‐2 since NEDD4‐2 was predicted and validated as the direct downstream target of miR‐454. NEDD4‐2 is increased in hypertrophied neonatal rat cardiomyocytes and participates in the development of HF by ubiquitinating Nav1.5.^11^ Besides, NEDD4‐2 with C2 domain has been reported to participate in the regulation of cardio‐renal syndrome in part through cardiac ion channels which are crucial for normal heart functions.^30^ Double‐stranded miRNAs are degraded to form single‐stranded mature miRNAs, which are subsequently assembled with Argonaute proteins to form RNA‐induced silencing complex (RISC). RISC acts on the 3'UTR of specific mRNAs to inhibit the translation process by means of directly degrading mRNAs.^31^, ^32^, ^33^ During our study, our data demonstrated that miR‐454 can bind NEDD4‐2 mRNA by dual‐luciferase reporter assay. Meanwhile, the rescue experiments revealed that restoration of NEDD4‐2 attenuated the protective effects associated with miR‐454 on H9c2 cells, indicating that miR‐454 exerts its functions by targeting NEDD4‐2.

Mechanistically speaking, our findings provided evidence indicating that NEDD4‐2 could ubiquitinate TrkA to promote its degradation through proteasomes in H9c2 cells, thereby downregulating TrkA expression. As previously reported, NEDD4‐2 bound to ion channels (I Na and I Kr), resulted in ubiquitination and degradation, and thus its isoform NEDD4‐2 C2‐induced electrophysiological impairment and triggered both cardiac conduction alterations and pro‐arrhythmic changes in post‐acute myocardial infarctions.^30^ Anta *et al* ^34^ concluded that TrkA is ubiquitinated by NEDD4‐2 in neuronal precursor cells under the mediation of ubiquitin‐specific protease 36, which was consistent with the findings of the current study. Previous research has also revealed that NEDD4‐2 can bind to TrkA via a PPXY motif leading to the ubiquitination and downregulation of TrkA in NGF‐dependent sensory neurons.^35^ The regulatory role of TrkA in cardiovascular diseases has been highlighted in previous research. TrkA has also been shown to prevent cardiomyocyte apoptosis^36^ and protect them against oxidative stress.^37^ In addition, the activation of TrkA/Akt pathway exerts protective functions against hypoxia/reoxygenation‐induced cardiomyocyte apoptosis.^38^ During the current study, NEDD4‐2‐induced TrkA ubiquitination in H9c2 cells to downregulate TrkA, a gene which exerts protection against H9c2 cell apoptosis and injury.

Furthermore, our data revealed that miR‐454 regulated the NEDD4‐2/TrkA/cAMP axis resulting in the suppression of H9c2 cells apoptosis and oxidative stress injury both in vitro and in vivo. Accumulating evidence continues to implicate cAMP pathway activation with the attenuation of HF.^39^ Diminished cAMP generation is regarded as a contributor to chronic HF with its relation to contractile dysfunction.^40^ A previous study emphasized reduced CREB as a responsible entity behind oxidative stress, ultimately facilitating the progression of HF by influencing cardiac growth and apoptosis.^41^ Increased formation of cAMP has been reported to be beneficial for chronic HF treatment by maintaining the TH1/TH2 phenotype.^42^ Additionally, the activation of the cAMP/PKA pathway and interaction with the GLP‐1 receptor have been reported to be cardioprotective in H9c2 cells exposed to hypoxia/reoxygenation.^43^ Our data suggested that the miR‐454/NEDD4‐2/TrkA axis activated cAMP pathway as evidenced by increased expression of cAMP pathway marker genes PKA and CREB, to protect against the apoptosis of H9c2 cells and subsequent myocardial damage. Evidence has previously been presented suggesting that hesperetin‐mediated TrkA could activate PKA and CREB in PC12 cells.^18^ Additionally, Karkoulias *et al* ^44^ asserted that TrkA transactivation acts to maintain the phosphorylation and activation of CREB in PC12 cells while Alexaki *et al* ^45^ also reported this interaction in microglia. These findings suggest the cardioprotective role of miR‐454 in HF in H9c2 cells was achieved by TrkA‐activated cAMP pathway.

**FIGURE 9:**
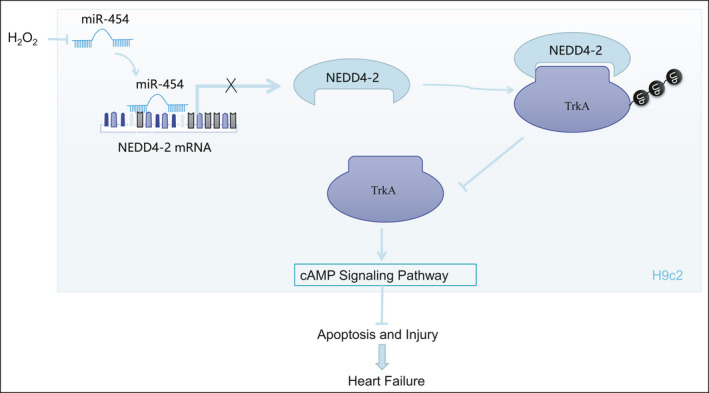
Schematic diagram displaying the mechanisms and cardioprotective implications of microRNA‐454‐mediated NEDD4‐2/TrkA/cAMP axis in HF

In conclusion, the current study provided evidence that miR‐454 elevation could target NEDD4‐2 to attenuate NEDD4‐2‐induced ubiquitination of TrkA and activate cAMP pathway for protection against H9c2 cell apoptosis and oxidative stress injury (Figure 9). These findings highlight the miR‐454‐mediated NEDD4‐2/TrkA/cAMP axis as a potential cardioprotective mechanism in progression of HF. Nevertheless, further validation through large‐scale samples and the use of primary neonatal cardiomyocytes is still required. Additionally, the cell type as the main source of circulating miR‐454 requires further investigation. In future investigations, we aim to explore the effects of miR‐454 inhibition, TrkA inhibition and NEDD4‐2 on the deterioration of cardiac function in non‐stress conditions and HF in vivo.

## CONFLICT OF INTEREST

The authors declare no conflicts of interest.

## AUTHOR CONTRIBUTIONS

**Yaowen Wang:** Conceptualization (equal); Investigation (lead); Validation (equal); Writing‐review & editing (equal). **Wei Pan:** Conceptualization (equal); Resources (equal); Software (lead); Writing‐original draft (equal). **Xinyu Bai:** Data curation (lead); Formal analysis (lead); Methodology (lead); Writing‐original draft (equal). **Xukai Wang:** Project administration (lead); Resources (equal); Writing‐review & editing (equal). **Yan Wang:** Conceptualization (equal); Writing‐original draft (equal). **Yuehui Yin:** Supervision (lead); Validation (lead); Writing‐review & editing (equal).

## Supporting information

Fig S1

Fig S2

Table S1

## Data Availability

The data that support the findings of this study are available from the corresponding author upon reasonable request.
