# Supplementary material for: microRNA‐454‐mediated NEDD4‐2/TrkA/cAMP axis in heart failure: Mechanisms and cardioprotective implications
Source: J Cell Mol Med. 2021 May 5;25(11):5082–98. doi: 10.1111/jcmm.16491 (PMC8178253; doi:10.1111/jcmm.16491)
Supplement: Supplementary file 3 — Table S1 [file JCMM-25-5082-s001.docx]

**Supplementary Table 1** Primer sequences for RT-qPCR

| Gene | Primer sequences |
| --- | --- |
| Human miR-454 | F: 5′-ACCCTATCAATATTGTCTCTGC-3′ |
|  | R: 5′-GCGAGCACAGAATTAATACGAC-3′ |
| Human U6 | F: 5′-CGCTTCGGCAGCACATATACTA-3′ |
|  | R: 5′-CGCTTCACGAATTTGCGTGTCA-3′ |
| Rat miR-454 | F: 5′-GGGACCCTATCAATATTGT-3′ |
|  | R: 5′-CAGTGCGTGTCGTGGAGT-3′ |
| Rat Nedd4-2 | F: 5′-ATGGAGCGACCCTATACATTTAAGGAC-3′ |
|  | R: 5′-TTAATCCACCCCTTCAAATCCTTGAGC-3′ |
| Rat TrkA | F: 5′-ACTAACAGCACATCAAGAGA-3′ |
|  | R: 5′-TCATTCAGAAGGTTGTAGCA-3′ |
| Rat U6 | F: 5′-CTTCGGCAGCACATATACT-3′ |
|  | R: 5′-AAAATATGGAACGCTTCACG-3′ |
| Rat GAPDH | F: 5′-TTCAACGGCACAGTCAAG-3′ |
|  | R: 5′-TACTCAGCACCAGCATCA-3′ |

Note: RT-qPCR, reverse transcription quantitative polymerase chain reaction; miR-454, microRNA-454; F, forward; R, reverse; Nedd4-2, neural precursor cell expressed, developmentally down-regulated 4-2; TrkA, tropomyosin receptor kinase A; GAPDH, glyceraldehyde-3-phosphate dehydrogenase.
